# 15 Traumatic Resections of the Hip-joint

**Published:** 1918-12

**Authors:** 


**15 Traumatic Resections of the Hip-Joint.** By MM. ALQUIER and TANTON. *Bulletin de la Société de Chirurgie de Paris*, May 28, 1918.

The 15 cases observed by the authors included : 1 resection beneath the head of the femur, 4 resections through the neck of the femur, 7 resections beneath the neck of the femur, 1 resection through the trochanter, and 4 resections beneath the trochanter.

4 were primary resections; 4 others, early secondary ones (2 to 7 days after injury); and 7, late secondary ones (10 to 42 days after injury, and even 6 months after, in one case). 3 deaths ensued, for patients were operated upon while suffering from severe infection and high fever. 2 of the operations were followed by suppurating arthritis in the corresponding knee. Arthrotomies were practised, and the knees healed, but remained ankylosed.

All the secondary interventions were necessitated by coxo-femoral suppurating arthritis or osteo-arthritis. In one case, however, no suppuration appeared, but the head of the femur presented osteomyelitis, and its resection was necessary. The authors think that primary resections undertaken after serious treatments of the wounds may have far better results for the joint than secondary ones.

The access to the joint is generally by way of the projectile's orifice. The authors practise as often as possible the Ollier's incision, or an external, vertical, incision. Immobilization is necessary after resections of the hip-joint. The authors accomplish it by means of a plastered apparatus, with extensible, metallic, splints; but this is fixed on only after 4 or 5 days, when the oozing has lessened. During the first 5 days, the limb is submitted to continuous extension. The resected limb is placed in abduction of 30 or 40° and external rotation.

